# Effects of climate on leaf phenolics, insect herbivory, and their relationship in pedunculate oak (*Quercus robur*) across its geographic range in Europe

**DOI:** 10.1007/s00442-025-05696-2

**Published:** 2025-04-05

**Authors:** Elena Valdés-Correcher, Yasmine Kadiri, Audrey Bourdin, Anna Mrazova, Flavius Bălăcenoiu, Manuela Branco, Michal Bogdziewicz, Mona Chor Bjørn, Thomas Damestoy, Jovan Dobrosavljević, Maria Faticov, Sofia Gripenberg, Martin M. Gossner, Maarten de Groot, Jonas Hagge, Jan ten Hoopen, Gabor L. Lövei, Slobodan Milanović, Dmitrii L. Musolin, Elina Mäntylä, Xoaquín Moreira, Andrea Piotti, Víctor M. Rodríguez, Cristina Saez-Asensio, Aurélien Sallé, Katerina Sam, Mar Sobral, Ayco J. M. Tack, Zulema Varela, Bastien Castagneyrol

**Affiliations:** 1 Integrative Ecology Group, Estación Biológica de Doñana, Seville, Spain; 2 Univ. Bordeaux, INRAE, BIOGECO, Cestas, France; 3 INRAE UE Ferlus, 86000 Lusignan, France; 4 Biology Centre of the Czech Academy of Sciences, Institute of Entomology, Branišovská 31, 370 05 České Budějovice, Czech Republic; 5 Faculty of Science, University of South Bohemia, Branišovská 1645/31a, 370 05 České Budějovice, Czech Republic; 6 National Institute for Research and Development in Forestry “Marin Drăcea”, Voluntari, Romania; 7 Forest Research Centre, Associate Laboratory TERRA, School of Agriculture, University of Lisbon, Lisbon, Portugal; 8 Forest Biology Center, Institute of Environmental Biology, Faculty of Biology, Adam Mickiewicz University, Uniwersytetu Poznańskiego 6, 61-614 Poznan, Poland; 9 Department of Geosciences and Natural Resource Management, University of Copenhagen, Rolighedsvej 23, 1958 Frederiksberg, Denmark; 10 UniLaSalle, AGHYLE, UP.2018.C101, FR-60026, Beauvais, France; 11 Faculty of Forestry, University of Belgrade, Kneza Višeslava 1, 11030 Belgrade, Serbia; 12 Département de Biologie, Université de Sherbrooke, Sherbrooke, QC Canada; 13 School of Biological Sciences, University of Reading, Reading, UK; 14 Forest Entomology, Swiss Federal Institute for Forest, Snow and Landscape Research WSL, Birmensdorf, Switzerland; 15 Institute of Terrestrial Ecosystems, Department of Environmental Systems Science, ETH Zürich, Zurich, Switzerland; 16 Department of Forest Protection, Slovenian Forestry Institute, Ljubljana, Slovenia; 17 Northwest German Forest Research Institute, Forest Nature Conservation, Prof.-Oelkers-Str. 6, 34346 Hann. Münden, Germany; 18 University of Göttingen, Forest Nature Conservation, Büsgenweg 3, 37077 Göttingen, Germany; 19 OneNature Ecology, Arnhem, the Netherlands; 20 Department of Agroecology, Aarhus University, Flakkebjerg ResearchCentre, 4200 Slagelse, Denmark; 21 HUN-REN-DU Anthropocene Ecology Research Group, University of Debrecen, 4010 Debrecen, Hungary; 22 Faculty of Forestry and Wood Technology, Mendel University in Brno, Zemedelska 3, 613 00 Brno, Czech Republic; 23 European and Mediterranean Plant Protection Organization (EPPO), 21 Boulevard Richard Lenoir, 75011 Paris, France; 24 Department of Biology, University of Turku, 20014 Turku, Finland; 25 Misión Biológica de Galicia (MBG-CSIC), Apartado de Correos 28, 36080 Pontevedra, Galicia, Spain; 26 Institute of Biosciences and BioResources, National Research Council of Italy, Sesto Fiorentino, Italy; 27 University of Orléans, Orléans, France; 28 Department of Geography, University of Santiago de Compostela, Praza da Universidade, 1, 15703 Santiago de Compostela, Spain; 29 Department of Ecology, Environment and Plant Sciences, Stockholm University, Stockholm, Sweden; 30 CRETUS, Ecology Unit, Department Functional Biology, Faculty of Biology, Universidade de Santiago de Compostela, 15782 Santiago de Compostela, Spain

**Keywords:** Leaf chemical defences, *Lymantria dispar*, Larvae biomass, Plant–insect interactions

## Abstract

An increase in biotic interactions towards lower latitudes is one of the most consistent patterns in ecology. Higher temperatures and more stable climatic conditions at low latitudes are thought to enhance biotic interactions, accelerating biological evolution and leading to stronger anti-herbivore defences in plants. However, some studies report contradictory findings, highlighting the need for further investigation into the underlying mechanisms. We used a combination of field observations and feeding trials in controlled environments to investigate the effect of climate on chemical defences and insect herbivory in pedunculate oak (*Quercus robur* L.) throughout most of its geographic range in Europe, while controlling for physical defences. The concentration of lignin, flavonoids, and total phenolics increased significantly with temperature, whereas both field herbivory and weight of spongy moth (*Lymantria dispar* L.) larvae were negatively influenced by temperature. Lignin concentration positively influenced the weight of spongy moth larvae whereas it had no effect on field herbivory. We found no evidence of strong positive relationships between insect herbivory and larvae growth with leaf defences. Our study underscores the complexity of plant–herbivore interactions along climatic gradients and highlights the need for further research to disentangle these intricate relationships.

**Supplementary Information:**

The online version contains supplementary material available at 10.1007/s00442-025-05696-2.

## Introduction

The increase of biotic interactions towards lower latitudes at both regional and continental scales is amongst the most ubiquitous patterns in ecology. Theory predicts that higher temperatures and more stable climatic conditions at low latitudes intensify biotic interactions and therefore accelerate the pace of biological evolution (Dobzhansky 1950; Janzen 1970; Schemske et al. 2009; Coelho et al. 2023). Within this framework, plants should have evolved stronger levels of anti-herbivore defences at lower latitudes where herbivore pressure is higher (Rasmann and Agrawal 2011; Pearse and Hipp 2012; Moreira et al. 2014; Abdala-Roberts et al. 2016). Thus, insect herbivory and consequently plant anti-herbivore defences should increase towards lower latitudes. This idea has ignited a passionate debate (Moles and Ollerton 2016; Anstett et al. 2016; Kozlov and Klemola 2017), fuelled by unsupportive results (Moles and Westoby 2003; Gaston et al. 2004; Moles et al. 2011) or even contrary to its predictions (Adams et al. 2009; del-Val and Armesto 2010; Woods et al. 2012; Moreira et al. 2018). The controversy requires further work to re-evaluate the predictions and test the underlying mechanisms.

There are some problematic assumptions regarding the variation in herbivory and plant defences along latitudinal gradients, which may explain the inconsistencies in empirical results. First, most previous studies have been conducted with groupings of herbivore species or guilds with different life histories that do not respond in the same way to biotic or climatic conditions and therefore, may have different relationships with latitude (for exceptions see Pennings et al. 2009; Salazar and Marquis 2012; Kim 2014; Anstett et al. 2014). In such cases, latitudinal clines in herbivory and plant defences may be confounded with changes in plant defences (Wetzel et al. 2016). Second, latitudinal clines represent complex gradients that encapsulate several factors that have direct effects on plants and insect herbivores. This is typically the case for temperature and precipitation, whose association with latitude may be obscured by topography, continentality or land use (De Frenne et al. 2013; Roslin et al. 2017; Loughnan and Williams 2019). Recent studies suggested that the latitudinal clines in herbivory and plant defences may have been biased by the unaccounted collinearity between latitude and its surrogates, such as temperature, precipitation and ecosystem complexity (biodiversity and vegetation type) (Loughnan and Williams 2019). Finally, herbivory is ultimately determined by the interplay between secondary metabolites that presumably act as chemical defences (e.g., phenolic compounds such as flavonoids or tannins), physical traits (e.g., leaf thickness and density, trichomes), and the disponibility of limiting macro-elements in plant organs, whereby these three covary in different ways and define plant defence syndromes (Caldwell et al. 2016). However, while numerous studies have examined the impact of chemical defences on insect herbivores across latitudinal gradients, only a few have effectively isolated the influence of chemical defences from other covarying factors, such as physical traits and nutrient levels (Więski and Pennings 2014; Demko et al. 2017) that also exhibit latitudinal variation (Andrew and Hughes 2005; Moles et al. 2011; Luo et al. 2019).

Several independent studies have addressed these limitations, but separately, thus overlooking potential interconnections. We fill this gap by using a combination of continent-wide field observations and feeding trials in controlled environments to investigate the effect of climate on leaf insect herbivory and chemical defences in pedunculate oaks (*Quercus robur* L.) while controlling for physical defences and nutrients. We measured herbivory and quantified secondary metabolites (phenolics) in 168 pedunculate oaks distributed across its European geographic range. We fed spongy moth larvae (*Lymantria dispar* L.) with a semi-artificial diet incorporating grounded leaves from the same oaks to isolate chemical traits from physical traits. Specifically, we asked: (1) How does climate influence leaf insect herbivory and chemical defenses in pedunculate oaks? (2) Does increased temperature lead to higher levels of chemical defenses (phenolics) in oak leaves? (3) How do these chemical defenses impact the performance and biomass of spongy moth larvae when physical traits are controlled for? (4) Does variability in field herbivory covary with the performance of spongy moth larvae? To address these questions, we predicted that (i) in the field, leaf insect herbivory and chemical defences would increase with increasing temperature. We also predicted that (ii) spongy moth larvae fed on oak leaves collected from trees in warmer environments would experience reduced growth (iii) as a result of higher amounts of chemical defences.

## Materials and method

### Study system

The pedunculate oak is a keystone species in many ecosystems and one of the dominant deciduous tree species in European forests with high ecological and economic importance (Eaton et al. 2016). This species experiences variable climatic conditions along its distribution range from central Spain (39 °N) to southern Fennoscandia (62 °N) (Petit et al. 2002). It is associated with a large and diverse community of generalist and specialist herbivorous insects (Brändle and Brandl 2001; Southwood et al. 2005; Marković and Stojanovic 2011; Moreira et al. 2018). Its widespread distribution and interactions with various herbivores make it a suitable representative for broader ecological studies. While the specific defence mechanisms may not directly translate to the defence mechanisms of other species, studying its interaction with insect herbivores along its distribution range can provide insights into fundamental ecological principles that may apply more broadly.

Oaks are associated with a large diversity of insect species, including primary and secondary pest species, with the spongy moth being a notable example. The spongy moth is a generalist polyphagous defoliator whose native range expands throughout Europe and Asia. It is able to feed on about 500 host species, including oaks. It causes large-scale damage to European forests during outbreaks (Liebhold and Elkinton 1988; Boukouvala et al. 2022). Its phenology is synchronized locally with that of oaks, so that egg hatch and bud burst typically occur at the same time, thereby causing major defoliation of oaks in spring and early summer (Wagenhoff et al. 2013). Although it can feed on many species, it does not develop equally well on all species and is best synchronized with oak, even when temperature will change in the face of climate change (Vitasse et al. 2024).

### Leaf samples

**Fig. 1:**
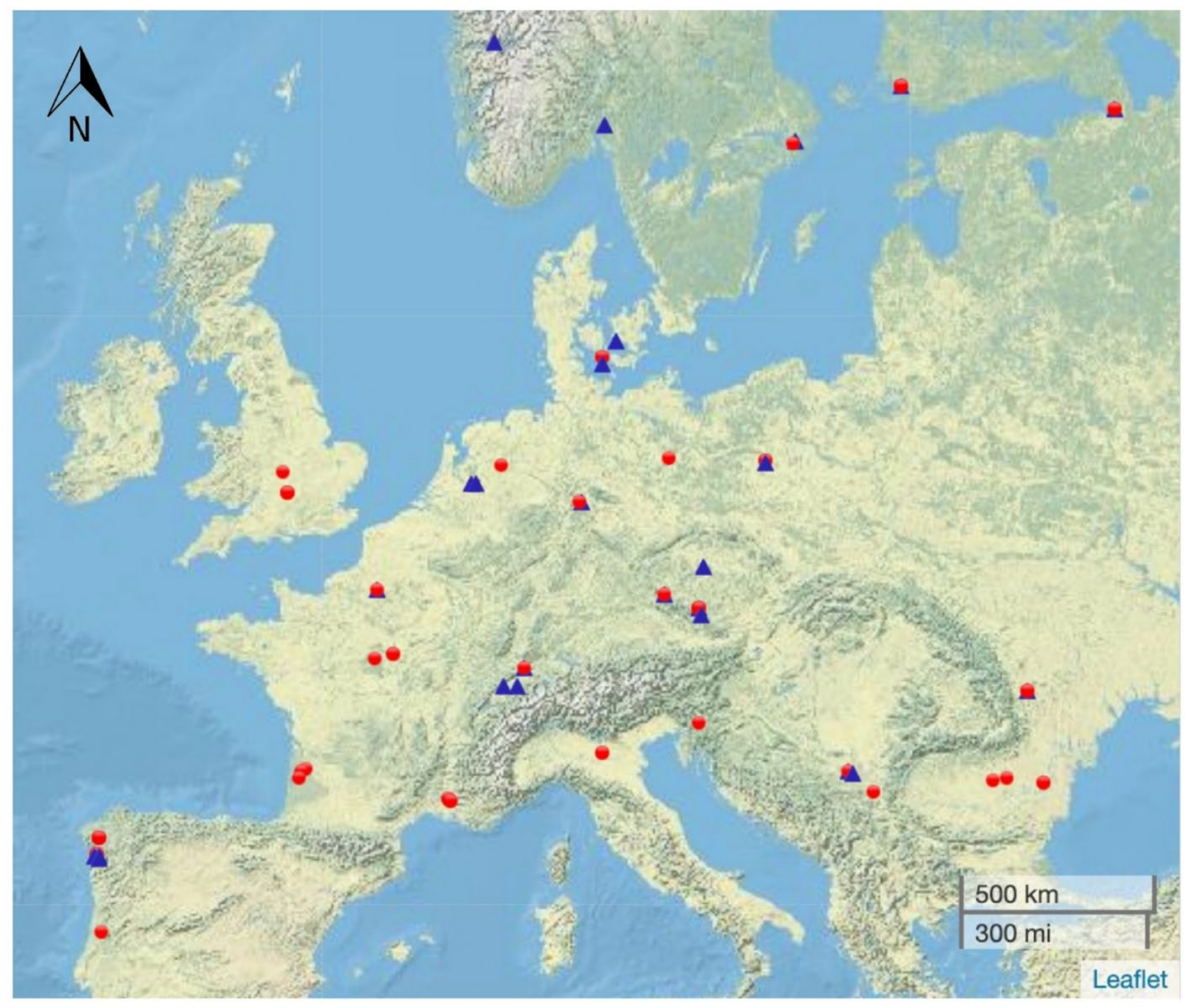
Locations of the trees sampled in 2020 (blue triangles) and 2021 (red circles). The map was produced using Leaflet (Cheng et al. 2021)

We sampled 168 pedunculate oaks in 2020 (25 sites) and in 2021 (34 sites) across 18 European countries (Fig. 1). At each site, consisting of woods or forests of 1 ha or more, we haphazardly selected three adult pedunculate oaks. We selected the focal oaks among those with low-hanging branches that could easily be reached from the ground.

**Fig. 2:**
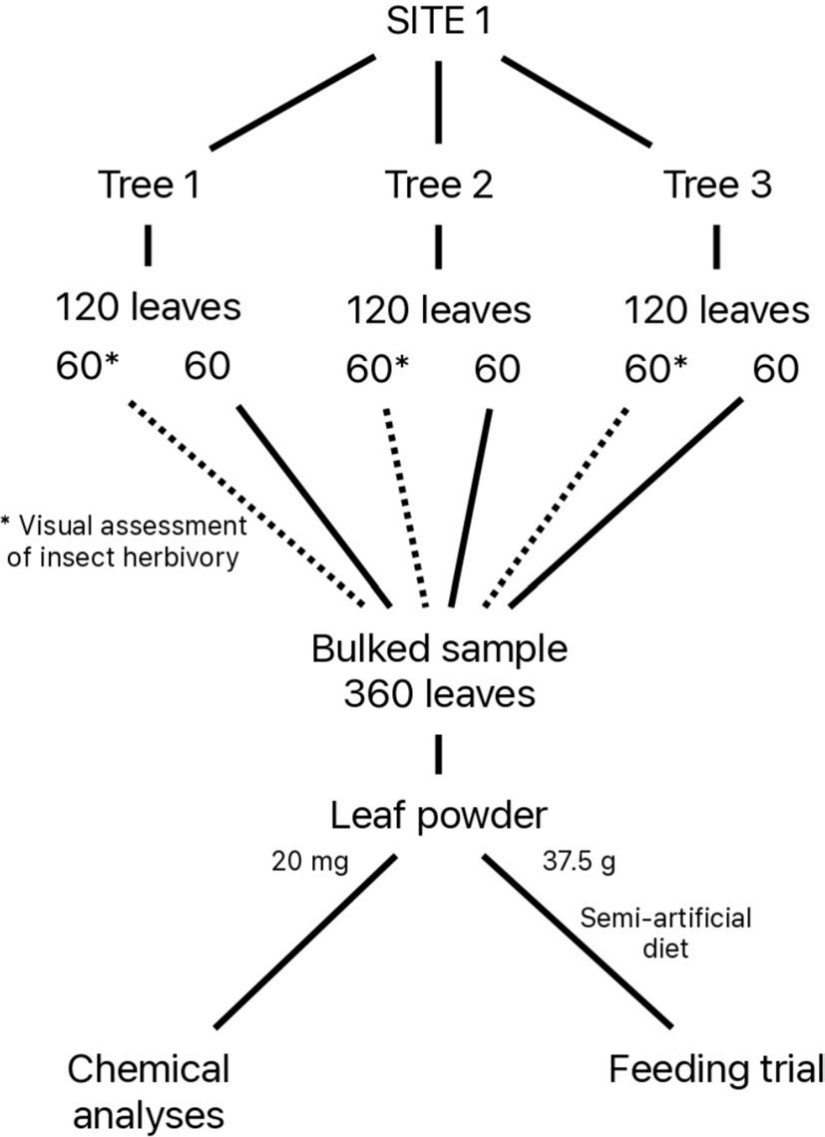
Schematic representation of leaf samples used for the quantification of leaf phenolics (chemical analyses), the assessment of herbivory and the laboratory feeding experiment (feeding trial)

In early summer—approximately 10–12 weeks after oak bud break at each site—we selected four low-hanging branches on each tree, pointing north, south, east and west directions, and haphazardly collected 30 developed leaves per branch, possibly at different time of the day, for a total of 120 leaves per tree (see Fig. 2). At this stage, leaves are fully expanded and mature, containing high levels of phenolic compounds such as tannins and flavonoids (Salminen et al. 2004). This period also coincides with peak herbivore activity, particularly that of caterpillars, which feed extensively on oak foliage (Southwood et al. 2004). Leaves were oven-dried 48 h at 45 °C immediately after collection, and sent to INRAE laboratory in Bordeaux (France) where they were stored for further chemical analyses of phenolic compounds. Although phenolic compounds are susceptible to oxidation during drying, previous trials demonstrated that their concentrations were similar between lyophilized frozen leaf samples and air-dried samples (see Fig. S1), suggesting that both methods provide comparable results in terms of phenolic content.

### Leaf phenolics

We quantified several leaf phenolics typically known as deterrents against insect herbivores in several oak species (Moreira et al. 2018). We extracted them from the mix of the 120 grounded leaves coming from three tree replicates per site (see Fig. 2). Twenty milligrams of the grounded material were mixed with 1 mL of 70% methanol in an ultrasonic bath for 15 min. We centrifuged and subsequently transferred them to chromatographic vials. To perform the chromatographic analyses, we used an ultra-high-performance liquid chromatograph (UHPLC Nexera LC-30AD; Shimadzu Corp., Kyoto, Japan) equipped with a Nexera SIL-30AC injector and one SPD-M20A UV/VIS photodiode array detector. Compound separation was carried out on a Kinetex 2.6 µm C18 82–102 Å, LC Column 100 × 4.6 mm (Phenomenex, Torrance, CA, USA), protected with a C18 guard cartridge. The flow rate was 0.4 mL min^−1^, and the oven temperature was set at 25 °C. The mobile phase consisted of two solvents: water-formic acid (0.05%) (A) and acetonitrile-formic acid (0.05%) (B), starting with 5% B and using a gradient to obtain 30% B at 4 min, 60% B at 10 min, 80% B at 13 min and 100% B at 15 min. The injection volume was between 15 and 30 µL. For phenolic compound identification, we used an ultra-performance liquid chromatograph coupled with an electrospray ionization quadrupole (Thermo Dionex Ultimate 3000 LC; Thermo Fisher Scientific, Waltham, MA, USA) time-of-flight mass spectrometer (UPLC-Q-TOF-MS/MS; Bruker Compact, Bruker Corp., Billerica, MA, USA).

We identified three groups of phenolic compounds: flavonoids, ellagitannins and gallic acid derivates (*hydrolysable tannins* henceforth) and hydroxycinnamic acid precursors to lignins (*lignins* henceforth). We quantified flavonoids as rutin equivalents, hydrolysable tannins as gallic acid equivalents, and lignins as ferulic acid equivalents (Moreira et al. 2018). We obtained the quantification of these phenolic compounds by external calibration using calibration curves at 0.25, 0.5, 1, 2 and 5 μg mL^−1^. Phenolic compound concentrations were expressed in mg·g-1 tissue on a dry weight basis.

We also quantified phenolic compound diversity at the individual plant level using two indices: phenolic compound richness, defined as the total number of phenolic compounds, and the Shannon diversity index. The Shannon diversity index was calculated as *H* = –Σ(*P*_*i*_ log[*P*_*i*_]), where *P*_*i*_ represents the relative abundance of a given phenolic compound, divided by the total phenolic content in each plant. Both of these indices are important for assessing a plant’s resistance to biotic factors, such as herbivores and pathogens (Wetzel and Whitehead 2020; Defossez et al. 2021). A higher number of unique phenolic compounds indicates a more diverse array of chemical defences, which can provide increased protection against herbivory (Wetzel and Whitehead 2020).

### Leaf herbivory

We assessed herbivory on a subset of 60 leaves per tree, blindly drawn from the original set of 120 leaves. These 60 leaves, along with the remaining 60 (unassessed) leaves were kept in a sealed plastic box with silica gel until they were processed for leaf phenolics and the feeding trial (see Fig. 2).

Leaf herbivory was visually scored by assigning each leaf to one of the following classes: 0, 0.1–5.0, 5.1–10.0, 10.1–15.0, 15.1–25.0, 25.1–50.0, 50.1–75.0 or > 75%, where the percentage represented the proportion of leaf surface removed by chewing herbivores or mined by leaf miners (Valdés-Correcher et al. 2021, 2022). We then used the midpoint of each percentage class to average herbivory at the site level. To minimise unconscious bias, herbivory was scored by a single trained observer (YK) who was unaware of leaf origin.

### Laboratory feeding experiment

We prepared a semi-artificial diet by assembling a commercial diet used for the rearing of lepidopteran species (Hervet et al. 2016) with leaf powder obtained by grinding the 120 dried leaves per tree (see Fig. 2). Note that part of this leaf powder was used for the extraction of leaf phenolics. We pooled the powder from the three tree replicates per site to obtain enough material to prepare the diet. The diet was obtained from the Insect Production and Quarantine Laboratories at the Great Lakes Forestry Centre (1219 Queen St E., Sault Sainte Marie, ON P6A2, Canada). One liter of it was obtained by mixing 17.36 g agar, 35 g casein, 5 ml 4 M KOH, 5 of alphacel, 10 g Wesson’s salt, 35 g sucrose, 30.69 g toasted wheat germ, 1 g choline chloride, 4 g ascorbic acid, 0.5 ml 37% formalin, 1.5 g methyl paraben, 2.1 g aureomycin, 5 ml linseed oil and 10 ml vitamin in 840 mL of distilled water. We added 37.5 g of grounded leaf material per liter to obtain a thick paste that was divided into 2 cm^3^ portions. Diet items therefore had the same basic composition with standard primary nutrients across different sites, but varied in a way that was representative of differences between sites in average amounts of chemical defence compounds, independent of differences in physical defences. The detailed procedure for the preparation of the diet is reported in the detailed method description of the Supplemental Material.

We obtained spongy moth larvae from egg masses collected on mature *Q. robur* in the wild in SE France. We fed larvae with fresh oak leaves collected on a single pedunculate oak in front of the laboratory, until they reached the 3rd instar. The rearing and experiments were conducted in a climatic chamber at constant temperature (22 °C) and relative humidity (40%) with a 12 h light: 12 h dark photoperiod.

One day before the start of the experiment, we isolated groups of three larvae, corresponding to three replicates for each sample site. We introduced larvae in separate vials and kept them without food for 24 h after which they were weighed individually to the closest 10 µg. We placed each larva into individual 24 cl paper cups with a portion of artificial diet and sealed the cups with a piece of organza fabric. We therefore obtained three rearing cups per sampled site. We renewed the diet pieces every two days and randomized the position of the cups in the climatic chamber after each replacement. After 7 days, to prevent weight measurement bias caused by undigested food, larvae were left without food for 24 h and then weighed individually (Castagneyrol et al. 2018).

The experiment was performed in 2021 and repeated 2022 with oak leaves sampled in 2020 and 2021, respectively. In total, it included 177 larvae that were reared on an artificial diet prepared from oak leaves from 55 sites (4 of the sites were sampled twice in 2020 and 2021). Fifty larvae died during the experiment, reducing the final sample size down to 127 larvae (74 fed leaves from 25 sites sampled in 2020 and 53 from 34 sites sampled in 2021). The dying larvae were a random occurrence and not correlated with any of the site factors.

### Statistical analysis

All analyses were conducted in R (R Core Team 2024) with packages *MuMIn* (*model.avg* and *dredge* functions) (Barton 2009) and *lme4* (*lmer* function) (Bates 2016). We analysed three response variables, using separate models that were all built comparably.

We analysed the amount of leaf phenolics (*leaf trait models* henceforth) (as total phenolics, phenolic compound richness and Shannon diversity index or for each of the three groups of phenolics separately—lignins, flavonoids, hydrolysable tannins) and leaf herbivory (*herbivory model*) with data aggregated at site level using linear models. We analysed the growth rate of spongy moth larvae (*growth model*) using Linear Mixed-effect Model (LMMs) with site as a random factor and modelled the growth of spongy moth larvae as their final weight, with initial weight as a covariate (Castagneyrol et al. 2018). The random factor was used to account for the non-independence of measurements made on three larvae fed the same diet. Each model was built with the following predictors (fixed effects): year (as a two-levels factor, 2020 vs. 2021), mean annual temperature (called temperature henceforth) and mean annual total precipitations (called precipitation henceforth) (as extracted from the *wordclim* database with a spatial resolution of 5 min, i.e., about 9 km at the equator on the basis of the tree coordinates [https://www.worldclim.org/], see Valdés-Correcher et al. 2021 for details). In both herbivory and growth models, we added leaf phenolics as fixed effects, considering total phenolics, phenolic compound richness, Shannon diversity index, flavonoids, lignins and hydrolysable tannins in separate models to avoid spurious estimates of model coefficients caused by collinearity among predictors. We scaled and centered all continuous predictor variables prior to modelling with the function *scale* from R to make their coefficients comparable, and verified that uncontrolled correlations among explanatory variables were unlikely to bias model coefficient parameter estimates (all variance inflation factors lower than 2) (Schielzeth 2010). Note that in our initial models, we included the proportion of attacked leaves as a fixed effect to account for its potential impact on the dependent variables. However, subsequent analyses showed that the inclusion of this covariate did not qualitatively or quantitatively alter the model outputs. Consequently, we removed the proportion of attacked leaves from the final models for sake of parsimony and to simplify the interpretation of the results. Although latitude was negatively and strongly correlated with temperature (Pearson *r* = − 0.87,* p* < 0.001, Table S1) and negatively but weakly correlated with precipitation (Pearson *r* = − 0.31, *p* < 0.05 Table S1), which could have led to collinearity issues, a previous study found that climatic variables were better predictors of variation in herbivory and leaf phenolics. Therefore, we chose to include only climatic variables in the models (Valdés-Correcher et al. 2021).

In total, we built four trait models, four herbivory models and four growth models (Table S2). We compared the different herbivory models and the different growth models separately in the framework of information theory (Burnham and Anderson 2002). We applied a procedure of parsimonious model selection based on the Akaike's Information Criterion corrected for small sample sizes (AICc) and considered every model in a range of 2 units of AICc to be the best model as equally likely (Arnold 2010). We calculated the AICc weights for each model (*w*_*a*_)—i.e., the probability that a given model is the best model within the set of candidate models—and also the relative variable importance (RVI), which reflects the importance of a particular variable in relation to all other variables, as the sum of *w*_*a*_ of every model including this variable. When multiple models were competing with the best model (i.e., several models have a ΔAICc < 2), we implemented a multi-model inference approach, constructing a consensus model that comprised the selected variables from the set of best models. We subsequently averaged their effect sizes over all models in the set of best models, utilising *w*_*a*_ as the weighting parameter (i.e., model averaging). We considered that a given predictor had a statistically significant effect on the response variable when its confidence interval did not bracket zero.

Eventually, we averaged field herbivory across the three tree replicates as well as specific relative growth rate (RGR) across the three larvae replicates, for each year separately. We calculated RGR of each larvae using Eq. 1:

1 $${\text{RGR}} = {1}00 \times \left( {w_{f} - w_{i} } \right)/w_{i}$$

where *w*_*i*_ and *w*_*f*_ are larvae initial and final weights, respectively. We correlated site-specific herbivory with site RGR.

## Results

### Leaf trait models

**Fig. 3:**
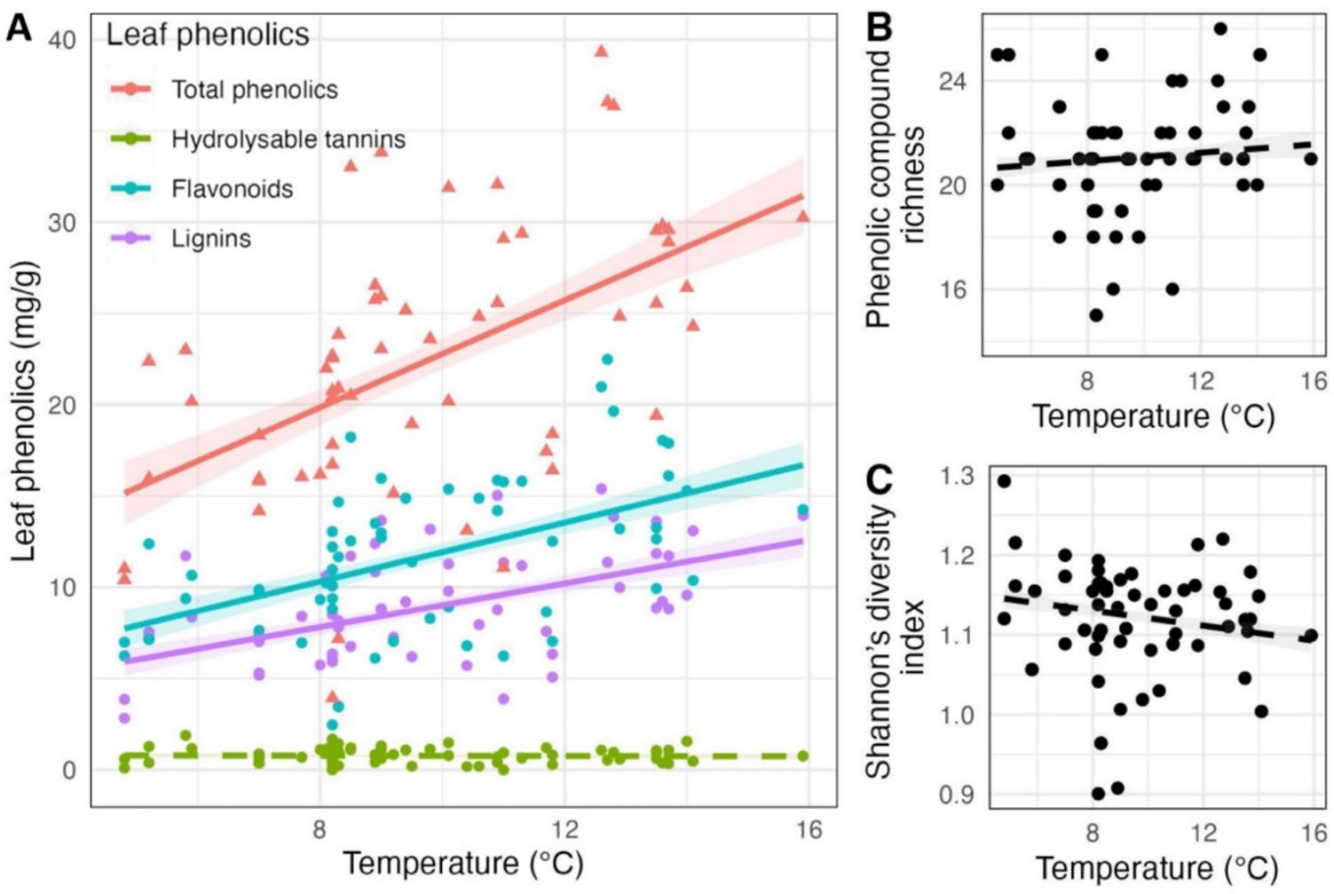
Effects of temperature on leaf phenolics (**A**: total phenolics, hydrolysable tannins, flavonoids and lignins; **B**: phenolic compound richness; **C**: Shannon diversity index). Triangle and circle shapes represent sites and correspond to total phenolics and to each of the three groups of phenolics and diversity index, respectively. Solid lines represent significant model predictions, while dashed lines represent non-significant model predictions in the linear models. Shaded areas correspond to standard errors

**Fig. 4:**
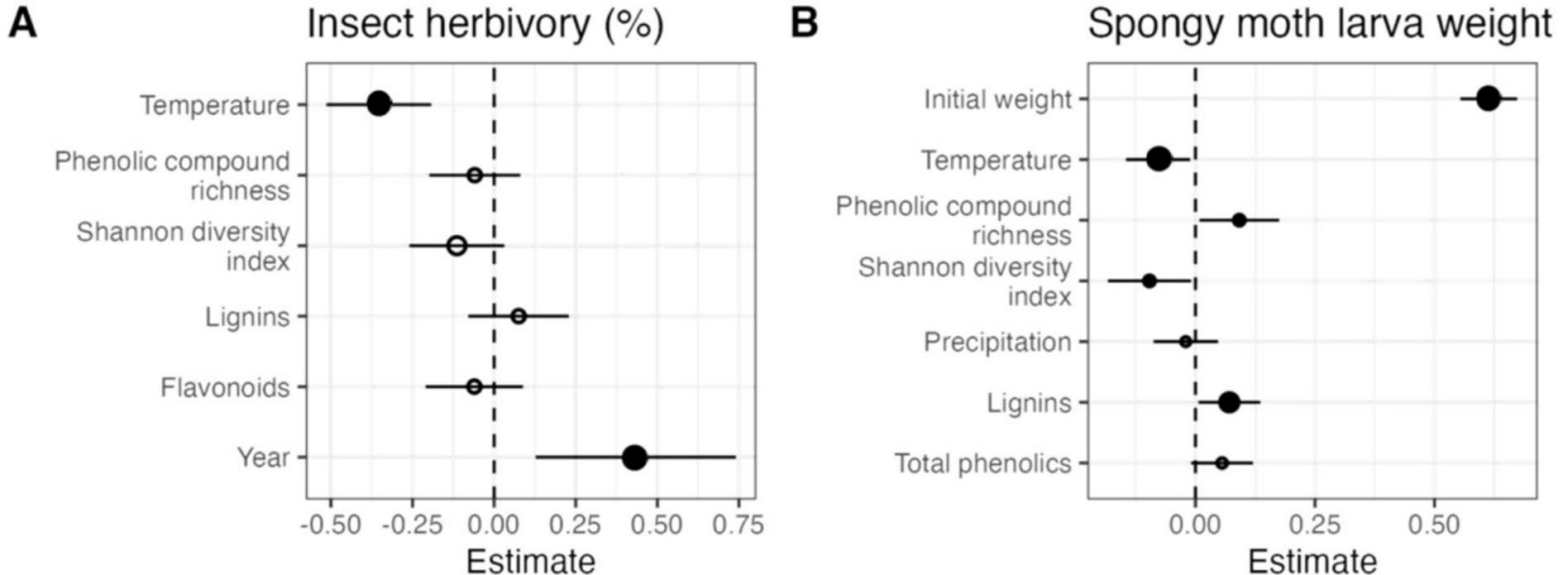
Effects of temperature and/or precipitation, leaf defenses (phenolic compound richness, Shannon diversity index, lignins, flavonoids and/or phenolic concentration), initial weight of the spongy moth larvae (note that initial weight was present only in the model of spongy moth weight) and year **A** on insect herbivory under natural conditions and **B** on final spongy moth larval weight under laboratory conditions. Note that only the variables retained in the selection of models are represented. Circles and error bars represent standardised parameter estimates and correspond to 95% confidence intervals (CI), respectively. The vertical dashed line centered on zero represents the null hypothesis. Full and empty circles represent significant and non- significant effect sizes, respectively. Circle size is proportional to the relative variable importance (RVI). The year 2020 is the intercept and was contrasted with the year 2021

The lignin concentration of the leaves averaged (± SE) 8.9 ± 0.4 mg/g, the flavonoids 11.7 ± 0.6 mg/g, the hydrolysable tannins 0.8 ± 0.1 mg/g, the phenolic concentration averaged 22.4 ± 1.1 mg/g, phenolic compound richness averaged 20.9 ± 1.0, while the Shannon’s diversity index averaged 1.12 ± 0.01. The concentration of lignins (Coef. ± SE = 1.15 ± 0.420, *p* = 0.008, *R*^*2*^ = 0.295), flavonoids (Coef. ± SE = 2.25 ± 0.581, *p* < 0.001, *R*^*2*^ = 0.251) and total phenolics (Coef. ± SE = 3.42 ± 0.977, *p* < 0.001, *R*^*2*^ = 0.295) increased significantly with temperature, while phenolic compound richness, Shannon diversity index, and the concentration of hydrolysable tannins did not vary with temperature (Figs. 3 and 4). The Shannon’s diversity index and the concentrations of hydrolysable tannins were higher in 2020 than in 2021 (Coef. ± SE = − 0.05 ± 0.019, *p* = 0.022, *R*^2^ = 0.116 for H, Coef. ± SE = − 0.58 ± 0.092, *p* < 0.001, *R*^2^ = 0.478 for the concentration of hydrolysable tannins), while the other leaf phenolics and S did not differ between years. None of the classes of leaf phenolics nor the Shannon diversity index varied with precipitation (Table S3).

### Herbivory model

**Fig. 5:**
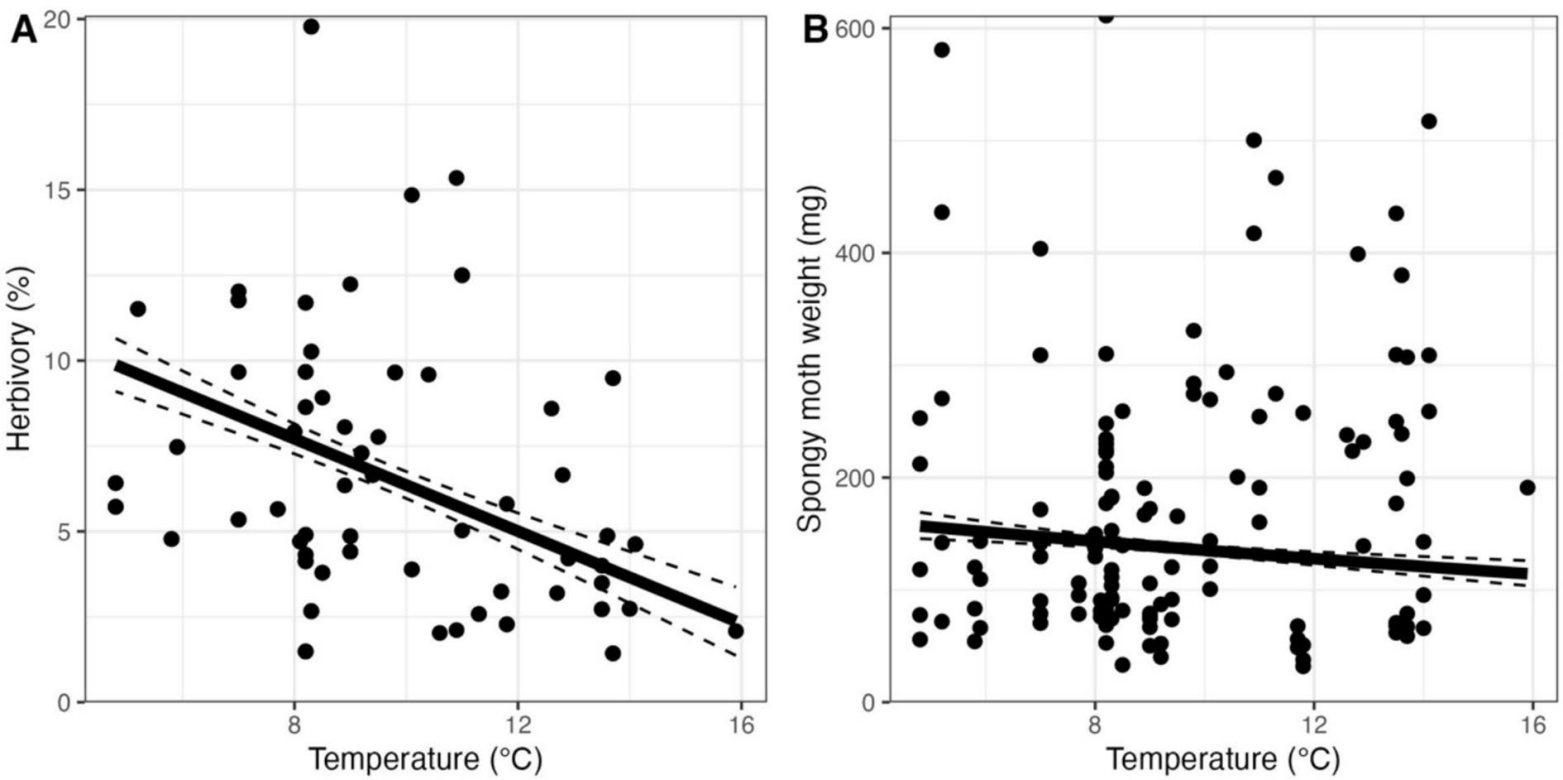
Effect of temperature on herbivory (**A**) and spongy moth weight (**B**). Solid and dashed lines represent model predictions and corresponding standard errors, respectively. The dots in panel A represent the average herbivory damage, calculated from 60 leaves analyzed per tree and averaged across each site, while dots in panel B correspond to the spongy moth weight of each larva (with 3 replicates per site). Only statistically significant relationships are shown

Under natural conditions, herbivores damaged on average (± SE) 7.1 ± 0.6% of the leaf area. Model selection retained models that included temperature (RVI = 1.00), phenolic compound richness (RVI = 0.13), Shannon diversity index (RVI = 0.32), lignins (RVI = 0.14), flavonoids (RVI = 0.12) and year (RVI = 1.00) as predictors explaining variability in herbivory (Fig. 4A; Table S4). Insect herbivory in the field decreased significantly with increasing site temperature (Coef. ± SE = − 0.37 ± 0.092, *p* < 0.001) (Fig. 5A) and it was on average significantly higher in the samples collected in 2021 than in the samples collected the previous year (0.40 ± 0.167, *p* = 0.002, *R*^2^ = 0.327) (Fig. 4A). Neither phenolic compound richness, Shannon diversity index, lignins nor flavonoids had significant effects on insect herbivory and had a low relative importance (Fig. 4A–C). Although not retained in the selection of models, hydrolysable tannins also had no significant effect on insect herbivory.

Spongy moth larvae gained on average (± SE) 21.69 ± 1.77 mg per day during the course of the experiment, representing an average relative growth rate of 49.6%. Model selection retained models that included initial larval weight (RVI = 1.00), temperature (RVI = 1.00), precipitation (RVI = 0.17), phenolic compound richness (RVI = 0.21), Shannon diversity index (RVI = 0.21), lignin concentration (RVI = 0.60) and phenolic concentration (RVI = 0.19) as predictors (Fig. 4B; Table S4). Final weight was positively influenced by initial weight (Coef. ± SE = 0.62 ± 0.029, *p* < 0.001) and lignin concentration (Coef. ± SE = 0.08 ± 0.033, *p* = 0.028, (Fig. 4B), but negatively influenced by the site temperature from which the oak leaves came from (Coef. ± SE = − 0.08 ± 0.037, *p* = 0.032, *R*^*2*^ = 0.844) (Figs. 4B and 5B). Neither precipitation nor total phenolic concentration had significant effects on larval final weight and had the lowest relative importance (Fig. 4B).

Although phenolic compound richness and Shannon diversity index showed significant effects on final weight in the model selection analysis, their low relative importance (RVI < 0.4) suggests that they are not consistent predictors in explaining the response variable and may have a marginal or context-dependent effect rather than a strong and consistent influence on the response variable.

Despite consistent responses to environmental variables, field herbivory was not correlated with RGR in laboratory conditions (Pearson's *r* = − 0.01, *P* = 0.928).

## Discussion

We tested three predictions regarding the relationship between temperature, herbivory, and plant defences in pedunculate oaks. Contrary to our first prediction, field insect herbivory decreased as temperature increased. Consistent with our expectations, we found that the concentrations of chemical defences (specifically, phenolics, flavonoids, and lignins) increased with rising temperatures. Supporting the second prediction, spongy moth larvae fed on oak leaves from warmer sites exhibited reduced biomass. The third prediction was partially supported: while temperature influenced larval biomass, the direct negative impact of phenolic concentrations on larval weight was not significant, and lignin concentration unexpectedly positively influenced larval biomass. Our combination of field observations and laboratory feeding trials brings new insights into the causes of large-scale variability in plant–herbivore interactions along climatic clines.

Contrary to our predictions, we found that both insect herbivory on oak leaves measured in the field and the performance of spongy moth larvae fed on diet containing these oak leaves in the laboratory decreased with increasing temperature at the European scale. As pointed out in the introduction, there is a growing controversy about the patterns and processes of large-scale variability in insect herbivory (Moles et al. 2011; Moles and Ollerton 2016; Zvereva et al. 2024). Mechanisms remain elusive, with no unequivocal proof that bottom-up forces—those driven by factors at the lower levels of the food chain, such as leaf phenolics affecting insect herbivores (Adams et al. 2009; Valdés-Correcher et al. 2021)—or top-down forces—those driven by predators or other factors at higher levels of the food chain affecting herbivores (Valdés-Correcher et al. 2021)—determine observed variability in herbivory. In the present study, we cannot completely rule out the possibility that reduced herbivory in the field at higher temperature was primarily driven by a lower herbivore density or activity. However, this explanation goes against the general view that herbivore density, diversity and activity increase towards lower latitudes. It is further ruled out by the similarity between the patterns we observed in the field and in the laboratory. Our findings contribute to this ongoing debate and suggest that factors other than temperature might play roles in shaping herbivory patterns, such as difference in herbivore community composition or the interaction of multiple environmental variables.

Contrasting with patterns in herbivory, we found that the total concentration of phenolic compounds in oak leaves increased with increasing temperature at the European scale, which is in line with our first prediction. Phenolic compounds are generally considered as chemical defences reducing insect herbivory in oaks (Fenny 1970; Roslin and Salminen 2008; Castagneyrol et al. 2018 but see Zvereva et al. 2024). An appealing interpretation of our results would therefore be that higher concentrations of leaf phenolics at sites with higher temperatures would, in part, reduce both herbivore performance and leaf herbivory (i.e., bottom-up control). However, our results only partially support this view, as the effect of temperature on insect herbivory in the field and on the performance of spongy moth larvae in the laboratory remained significant after the effect of phenolic compounds was accounted for in statistical models. This suggests that other environmental factors, such as altitude, light intensity, or soil properties, which can influence both plant chemistry and herbivore dynamics, may also play a role in shaping herbivore patterns (Uyi 2020; Hu et al. 2021). Future studies should consider these additional climatic and edaphic variables to better understand the complex interactions driving insect herbivory at broad spatial scales. Moreover, temperature increases have also been linked to higher terpene concentrations in plants, which may further influence insect herbivory through their toxic or deterrent effects (Irving et al. 2023). In addition, contrary to our predictions, the concentration of lignins was positively associated with the increase on biomass of spongy moth larvae in the laboratory, and none of the leaf phenolics we quantified were associated with herbivory in the field. Although the phenolic compound richness was also positively associated with the increase on biomass of spongy moth larvae, while the Shannon diversity index showed a negative association, their importance was weak (RVI < 0.4), suggesting that neither factor are reliable predictors of the biomass gain of spongy moth larvae. This unexpected positive association between lignins and larval weight may arise because herbivores selectively feed on certain leaves or tissues. Higher lignin concentrations might be linked to other favourable traits, such as increased nitrogen content, in some oak varieties (Cuchietti et al. 2014). While lignins are generally considered deterrents to herbivores (Mithöfer and Boland 2012), their relationship with larval mass could be shaped by a combination of factors that balance plant defence with herbivore nutritional needs (Damestoy et al. 2019). Further research is needed to explore these interactions and clarify the role of lignins in herbivore performance.

Insect herbivory in the field included damage from both generalist and specialist leaf chewers and miners, which may interact differently with leaf metabolites (Valdés-Correcher et al. 2021). In contrast, our laboratory experiment used a generalist herbivore, which has the ability to adjust its gut enzyme production in response to the nutritional quality of its diet (Milanović et al. 2016). This allows generalist species to compensate for potential adverse effects of leaf phenolics (Lazarević et al. 2002; Lazarević and Perić-Mataruga 2003; Damestoy et al. 2019), at least on the short term. Furthermore, constitutive levels of leaf defences do not always represent a generalized response to long-term herbivore pressure. Instead, they can vary depending on the quality and accumulation of defences after herbivore attacks, reflecting inducible defences. The absence of a clear relationship between leaf phenolics and insect herbivory in the field suggests that other leaf traits may overshadow the effects of phenolic concentrations under natural conditions (Castagneyrol et al. 2018). This finding challenges the common assumption that leaf phenolics are primarily defensive compounds. Previous studies have also questioned the direct impact of phenolic compounds on insect herbivory, noting that these compounds are involved in various plant functions beyond defence, such as contributing to ultraviolet protection and heat tolerance (Close and McArthur 2002; Zvereva et al. 2024). For instance, certain phenolic compounds, such as anthocyanins, play a crucial role in photoprotection, while quercetin derivatives, which are ortho-diphenols, exhibit antioxidant properties (Naikoo et al. 2019; Agati et al. 2020).

Insect herbivory is strongly influenced by leaf structural traits such as leaf specific area, leaf dry matter content or leaf toughness (Pérez-Harguindeguy et al. 2003; Clissold et al. 2009; Loranger et al. 2012). Yet, although performed in different regions and at different scales, several authors reported latitudinal clines in the expression of these traits (Andrew and Hughes 2005; Moles et al. 2011; Luo et al. 2019), making them good candidates to explain large scale variability in herbivory. For instance, Graça and Cressa (2010) investigated the leaf quality at a larger scale including tropical and temperate tree species and found that leaves in the tropics were tougher than leaves in temperate zones and consequently leaf consumption by chewers was negatively correlated with leaf toughness. Possibly, the relationship between temperature and insect herbivory was driven by such structural traits that were negatively associated with herbivory but positively associated with temperature, or conversely by traits that promoted herbivory while being negatively influenced by temperature. Here, we disabled physical traits by incorporating leaf material into an artificial diet in the feeding trials. If physical traits are an important mechanistic link between temperature and herbivory, the effect of temperature as inferred from the feeding trial would have differed from that inferred from field observations. We therefore suggest that even if leaf traits varied across the range of the pedunculate oak in Europe, this variation did not explain the variability in herbivory observed in our study (Valdés-Correcher et al. 2021).

## Conclusion and perspectives

It is expected that higher herbivory pressure under warmer conditions leads to stronger plant defences. Our work challenges this expectation and, more generally, questions the overall role of phenolic compounds as defences against insect herbivores, despite evidence that some phenolic compounds are known to have defensive properties. Indeed, we found that both herbivory and the concentration of phenolic compounds covaried with temperature at the European scale within a single plant species, but we found no evidence for strong functional relationships between them. Consistency in patterns observed in the field and in the laboratory further suggests that physical traits were primarily responsible for the general decrease in herbivory with increasing temperature. It is however striking that the present results conflict with previous work on the same tree species. For instance, Moreira et al. (2018) reported an increase in the incidence of insect herbivory towards lower latitudes, while Valdés-Correcher et al. (2021) found no statistically clear effect of latitude or climate on insect herbivory in the same oak species. Valdés-Correcher et al. (2021) measured insect herbivory similarly but on both forested areas and isolated oaks, whereas Moreira et al. (2018) measured it as the proportion of leaves damaged by herbivores at the end of the growing season and it included leaf damage by chewers, leaf miners and gallers separately. We also found that insect herbivory and the concentration of leaf phenolics varied greatly between years and in opposite directions. A promising research question would be to decipher the effects of longer-term differences in regional climate from the effect of current-year climatic conditions on plant traits and insect herbivores, to measure individual phenolics and not aggregated measures of leaf phenolics and to measure different feeding guilds separately.

## Supplementary Information

Below is the link to the electronic supplementary material.

Supplementary file1 (DOCX 210 kb)

## Data Availability

The dataset used and/or analysed during the current study will be deposited in a repository (10.20350/digitalCSIC/17083) prior to the publication of this manuscript.

## Declarations

### Rights and permissions

For the purpose of Open Access, a CC-BY 4.0 public copyright licence (https://creativecommons.org/licenses/by/4.0/) has been applied by the authors to the present document and will be applied to all subsequent versions up to the Author Accepted Manuscript arising from this submission.
