# Supplementary material for: Effects of climate on leaf phenolics, insect herbivory, and their relationship in pedunculate oak (Quercus robur) across its geographic range in Europe
Source: Oecologia. 2025 Apr 5;207(4):61. doi: 10.1007/s00442-025-05696-2 (PMC11972190; doi:10.1007/s00442-025-05696-2)
Supplement: Supplementary file 1 — Supplementary file1 (DOCX 210 kb) [file 442_2025_5696_MOESM1_ESM.docx]

**
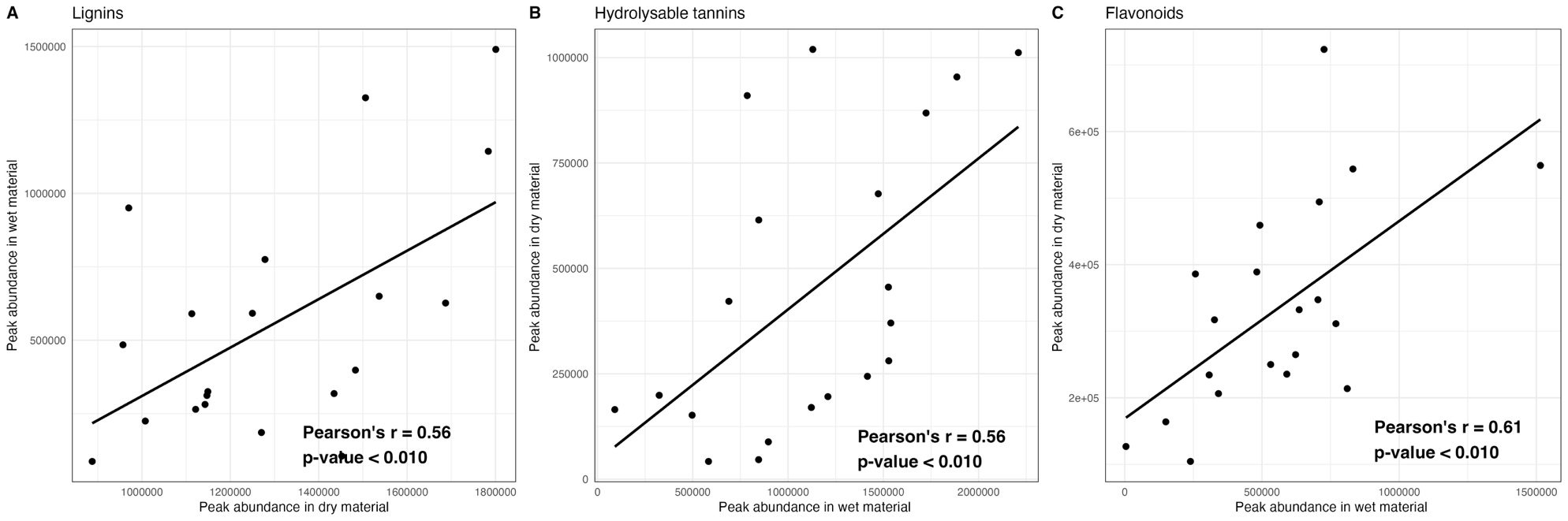
**

**Supplementary figure 1.** Correlations between the peak abundance of the three identified groups of phenolics—lignins, hydrolysable tannins, and flavonoids—measured in both lyophilized frozen leaf samples (x-axis) and dried leaf samples (y-axis) from 20 Quercus robur trees.

**Supplementary table 1.** Pearson correlation matrix showing the relationships between temperature, precipitation and latitude of the sites. Values represent correlation coefficients (r), with significance levels indicated by asterisks (**p* < 0.05, ***p* < 0.01, ****p* < 0.001).

| Variable | Temperature | Precipitation | Latitude |
| --- | --- | --- | --- |
| Temperature | 1.00 | 0.39* | -0.87^***^ |
| Precipitation |  | 1.00 | -0.31^*^ |
| Latitude |  |  | 1.00 |

**Supplementary table 2.** Summary of the response variables and fixed effects included in each model.

| **Models type** | **Response variables** | **Constant**  **fixed effects*** | **Separate   fixed effects**** | **Random  effects** |
| --- | --- | --- | --- | --- |
| Leaf trait model   (linear model) | Phenolic compound richness  Shannon diversity index  Total phenolics  Hydrolysable tannins  Flavonoids Lignins | Year   Temperature  Precipitation | - | - |
|  |  |  | - | - |
|  |  |  | - | - |
|  |  |  | - | - |
|  |  |  | - | - |
|  |  |  | - | - |
| Herbivory model   (linear model) | Insect herbivory (%) |  | Phenolic compound richness | - |
|  |  |  | Shannon diversity index | - |
|  |  |  | Total phenolics | - |
|  |  |  | Hydrolysable tannins | - |
|  |  |  | Flavonoids | - |
|  |  |  | Lignins | - |
| Growth model   (LMM) | Spongy moth   larva weight (g) |  | Phenolic compound richness | Site |
|  |  |  | Shannon diversity index | Site |
|  |  |  | Total phenolics | Site |
|  |  |  | Hydrolysable tannins | Site |
|  |  |  | Flavonoids | Site |
|  |  |  | Lignins | Site |

* Fixed effects present in models simultaneously

**Fixed effects added in separate models to avoid spurious estimates of model coefficients caused by collinearity among predictors

**Supplementary table 3.** Summary of linear models testing the effect of year, temperature and precipitation on leaf phenolics. Significant coefficients (*P* < 0.05) are in bold. Year is the effect of each year (2021 contrasted with 2020).

| **Response** | **Predictors** | ***F* value (df)** | ***P*-value** | **Estimate (SE)** | ***R2*** |
| --- | --- | --- | --- | --- | --- |
| Phenolic compound  richness | Year | 0.068 (1) | 0.796 | 0.208 (0.799) | 0.03 |
|  | Temperature | 0.552 (1) | 0.461 | 0.323 (0.435) |  |
|  | Precipitation | 0.219 (1) | 0.642 | 0.210 (0.448) |  |
| Shannon  diversity  index | Year | 5.562 (1) | **0.022** | -0.046 (0.019) | 0.12 |
|  | Temperature | 0.271 (1) | 0.604 | 0.006 (0.019) |  |
|  | Precipitation | 2.871 (1) | 0.0959 | -0.019 (0.011) |  |
| Total  phenolics | Year | 1.867 (1) | 0.177 | 2.454 (1.796) | 0.3 |
|  | Temperature | 12.212 (1) | **<0.001** | 3.416 (0.977) |  |
|  | Precipitation | 0.214 (1) | 0.646 | 0.466 (1.007) |  |
| Flavonoids | Year | 0.019 (1) | 0.89 | 0.149 (1.068) | 0.25 |
|  | Temperature | 15.056 (1) | **<0.001** | 2.254 (0.581) |  |
|  | Precipitation | 0.236 (1) | 0.629 | -0.290 (0.598) |  |
| Hydrolysable  tannins | Year | 39.502 (1) | **<0.001** | -0.582 (0.092) | 0.48 |
|  | Temperature | 0.522 (1) | 0.473 | 0.036 (0.050) |  |
|  | Precipitation | 3.620 (1) | 0.062 | 0.099 (0.052) |  |
| Lignins | Year | 1.955 (1) | 0.168 | 1.079 (0.772) | 0.3 |
|  | Temperature | 7.484 (1) | **0.008** | 1.149 (0.420) |  |
|  | Precipitation | 3.439 (1) | 0.069 | 0.802 (0.433) |  |

**Supplementary table 4.** Summary of model coefficient parameter estimates (i.e., effect sizes), degrees of freedom (df), log-likelihood, AICc, ΔAICc, AICc weight and the variance explained by fixed (*R^2^_m_*) and fixed plus random factors (*R^2^_c_*) of the leaf herbivory model (herbivory model, A) and the growth rate of spongy moth larvae model (growth model, B). Explanatory variables that cannot be included in the model selection for a given response variable are shaded. Those values are in bold if the variation of the response variable as a function of the explanatory variable is significant. Year is the effect of each year (2021 contrasted with 2020).

|  | **Phenolic compound  richness** | **Shannon  diversity  index** | **Total  phenolics** | **Flavonoids** | **Hydrolysable  tannins** | **Lignins** | **Precipitation** | **Temperature** | **Initial larval  weight** | **Year** | **df** | **logLik** | **AICc** | **∆AICc** | **weight** | ***R²_m_ (R²_c_)*** |  |
| --- | --- | --- | --- | --- | --- | --- | --- | --- | --- | --- | --- | --- | --- | --- | --- | --- | --- |
|  |  |  |  |  |  |  |  |  |  |  |  |  |  |  |  |  |  |
|  |  |  |  |  |  |  |  |  |  |  |  |  |  |  |  |  |  |
| A |  | -0.114 |  |  |  |  |  | **-0.356** |  | **+** | 5 | -44.836 | 100.8 | 0 | 0.147 | *0.33* |  |
|  |  |  |  |  |  |  |  | **-0.3519** |  | **+** | 4 | -46.126 | 101 | 0.19 | 0.134 | *0.33* |  |
|  |  |  |  |  |  | 0.0754 |  | **-0.3903** |  | **+** | 5 | -45.618 | 102.4 | 1.56 | 0.067 | *0.31* |  |
|  | -0.0505 |  |  |  |  |  |  | **-0.3426** |  | **+** | 5 | -45.738 | 102.6 | 1.8 | 0.06 | *0.31* |  |
|  |  |  |  | -0.0605 |  |  |  | **-0.3181** |  | **+** | 5 | -45.774 | 102.7 | 1.87 | 0.058 | *0.31* |  |
| B |  |  |  |  |  | **0.0695** |  | **-0.0833** | 0.6188 |  | 6 | -7.882 | 28.5 | 0 | 0.103 | *0.84 (0.89)* |  |
|  | **0.0917** | **-0.0961** |  |  |  |  |  | **-0.0689** | 0.5979 |  | 7 | -7.467 | 29.9 | 1.41 | 0.051 |  |  |
|  |  |  | 0.0572 |  |  |  |  | **-0.0783** | 0.6179 |  | 6 | -8.704 | 30.1 | 1.65 | 0.045 |  |  |
|  |  |  |  |  |  | **0.0747** | -0.0202 | **-0.0772** | 0.6147 |  | 7 | -7.705 | 30.4 | 1.89 | 0.04 |  |  |
